# Postoperative lymphopenia: An independent risk factor for postoperative pneumonia after lung cancer surgery, results of a case-control study

**DOI:** 10.1371/journal.pone.0205237

**Published:** 2018-10-15

**Authors:** Guillaume Dupont, Laura Flory, Jérôme Morel, Anne-Claire Lukaszewicz, Arnaud Patoir, Emilie Presles, Guillaume Monneret, Serge Molliex

**Affiliations:** 1 Département d’Anesthésie—Réanimation, Centre Hospitalier Universitaire de Saint-Étienne, Avenue Albert Raimond, France; 2 EA 7426 PI3—Pathophysiology of Injury-Induced Immunosuppression (Université Claude Bernard Lyon 1 / HCL / bioMérieux), Hôpital E., France; 3 Service d’Anesthésie Réanimation, Groupement Hospitalier Est, Hôpital Neurologique Pierre Wertheimer, France; 4 Département de chirurgie thoracique, Centre Hospitalier Universitaire de Saint-Etienne, France; 5 Inserm, CIC1408, France; 6 CHU Saint-Etienne, Hôpital Nord, Service Unité de Recherche Clinique, Innovation et Pharmacologie, Saint-Etienne, France; 7 Hospices Civils de Lyon, Laboratoire d’immunologie, Hôpital E Herriot, Lyon, France; University of South Alabama Mitchell Cancer Institute, UNITED STATES

## Abstract

**Objective:**

Postoperative lymphopenia has been proposed as a risk factor for postoperative infections but has never been identified as such in a multivariate analysis. Postoperative pneumonia (POP) is one of the most common complications after lung cancer surgery and is associated with a worse outcome. We aimed to evaluate the association between postoperative lymphopenia and POP after lung cancer surgery.

**Methods:**

Patients admitted for lung cancer surgery (lobectomy, bilobectomy, or pneumonectomy) aged ≥ 18 years and with no history of an immunosuppressive state were eligible for inclusion. Lymphocyte counts were determined in blood drawn on the day before surgery and at postoperative days 1, 3 and 7. POP diagnosis was based on clinical, biological and radiological data. A logistic regression model adjusted on currently described risk factors for POP was used to explain the onset of this condition.

**Results:**

Two hundred patients were included, of whom 43 (21.5%) developed POP. Preoperative lymphocyte count was 1.8±0.6x10^9^ cells/L and 2.0±0.7x10^9^ cells/L in patients with and without POP, respectively (*P* = .091). In both groups, the lymphocyte count nadir occurred at postoperative day 1. In multivariate analysis, lymphopenia at postoperative day 1 was significantly associated with increased risk of POP (odds ratio: 2.63, 95% CI [1.03–5.40]). POP rate at postoperative day 7 was higher in patients presenting low lymphocyte counts (≤1.19x10^9^ cells/L) at postoperative day 1 (*P* = .003).

**Conclusions:**

Our study showed that lymphopenia following lung cancer surgery was maximal at postoperative day 1 and was associated with POP.

## Introduction

Postoperative lymphopenia, reaching a nadir from two hours to two days after surgery, has been described for more than thirty years. As lymphocytes are a major component of infection control, postoperative lymphopenia has been proposed as a risk factor for postoperative infections [1]. However, the studies reporting postoperative lymphopenia were heterogeneous regarding surgical procedures (abdominal, thoracic, neurologic, etc.) or inclusion of patients with cancer, and were restricted to a single center. All included small patient populations and did not take into account the currently recognized risk factors for postoperative infection, thereby creating a confounding effect.

Postoperative pneumonia (POP) is one of the most common complications after lung cancer surgery with a reported incidence ranging from 9 to 25% [2,3]. POP is associated with a worse outcome, including increased long-term mortality, prolonged hospital stay and a significant increase in hospital care costs [4–6]. To prevent POP, it is essential to identify the relevant risk factors.

We hypothesized that post-operative lymphopenia was an independent risk factor for POP. Our primary objective was to evaluate the association between postoperative lymphopenia and POP after lung cancer surgery. Secondary objective was to precise the time to lymphopenia nadir.

## Material and methods

### Study design

This was a case-control, single-center study, conducted in the University Hospital of Saint Etienne and approved by our local ethics committee (*Comité d’éthique N° IRBN172017/CHUSTE*, *CNIL No*. *2028629)*. Informed consent was deemed not to be required.

Data were obtained from a surgical database made between January 2013 and May 2015. Patient file were screened retrospectively. Patients were monitored until discharge from the hospital, or up to postoperative day 7. If an infection occurred before hospital discharge or before postoperative day 7, monitoring was discontinued.

Lymphocyte counts were obtained from total blood counts routinely performed prior to surgery and during the postoperative period. The lymphocyte counts analyzed in this study were those determined in blood drawn on the day before surgery and at postoperative days 1, 3 and 7.

Patients over 18 years of age admitted for lung cancer surgery (lobectomy, bilobectomy, or pneumonectomy) were eligible for inclusion. Exclusion criteria comprised pre-operative infections, patients with no respect of French antibiotic prophylactic and for which antibiotic prophylactic were continued longer than recommended, HIV infection, history of malignant blood disease, autoimmune disease, immunosuppressive treatment or corticosteroid treatment at doses exceeding 10 mg/day prednisolone or equivalent, history of radiotherapy or chemotherapy within the last 6 months, and new organ failure (SOFA score ≥ 2 or increase ≥ 2) after surgery.

### Outcome

The primary end point was the occurrence of POP before postoperative day 7 and its association with postoperative lymphocyte count.

Clinical data, including comorbidities, type of surgical resection, anesthetic procedures, and length of hospital stay were recorded. Data were collected concerning six risk factors for POP currently described in the literature, namely sex, age > 65 years, American Society of Anesthesiologists (ASA) score ≥ 3, chronic obstructive pulmonary disease (COPD), active smoking or past smoking (stopped less than 8 weeks prior to inclusion), and chronic heart failure (NYHA ≥ 2) [2,4–8].

### Definition of postoperative pneumonia

As previously described, diagnosis of POP required patients to meet two major criteria plus one minor criterion, or one major criterion plus three minor criteria as listed below [2,4]:

- Major criteria: fever > 38°C with no other recognizable cause, abnormal radiographic finding (new or progressive and persistent infiltrate, consolidation or opacity).
- Minor criteria: leukopenia < 4.0 x 10^9^ cells/L or leukocytosis ≥ 12.0 x 10^9^ cells/L, new rise in C-reactive protein level, increase and modification of the expectorate toward a purulent appearance.

Three experienced clinicians reported the chest X-rays: two intensive care specialists and a cardiothoracic surgeon. All information needed for POP diagnosis were obtained from patient file.

### Statistical analysis

We calculated a sample size of 175 patients considering 6 risks factors for POP currently known (supposed to be independent) plus the nadir of postoperative lymphocyte count, a POP rate of 20% and five patients per variable tested [9–12]. Considering that, 35 patients with POP and 140 with no POP were expected.

The results were expressed as percentages or as mean values ± SD. Values of continuous variables determined preoperatively and at the time of the lymphocyte nadir were compared using a nonparametric test (Mann-Whitney test). Qualitative variables were compared using a Chi2 test. A Cox model was used to explain POP. This model aimed to predict postoperative lymphopenia as a risk factor for POP. It was adjusted on currently described risk factors for POP, namely age > 65 years, sex, ASA score ≥ 3, COPD, active smoking and chronic heart failure (NYHA ≥ 2) [2,4–8]. The Hazard ratio and associated 95% confidence interval were determined for each variable linked to lymphocyte count. To estimate the discriminating power of postoperative lymphopenia for the diagnosis of POP, receiver operating characteristic (ROC) curves were plotted and areas under the curve were calculated. Sensitivity, specificity, and positive and negative predictive values were calculated at the cutoff value of lymphopenia for the diagnosis of POP. Kaplan-Meier curves were generated for POP using the cutoff value of lymphopenia determined from the ROC curves. Missing datas were taken into account in the analysis.

Statistical analyses were conducted using SAS-Windows^®^ (SAS Institute Inc., Campus Drive Cary, USA) Version 9.4 software and XLSTAT (Addinsoft, Paris, France) for Mac Version 19.1.

## Results

**Fig 1:**
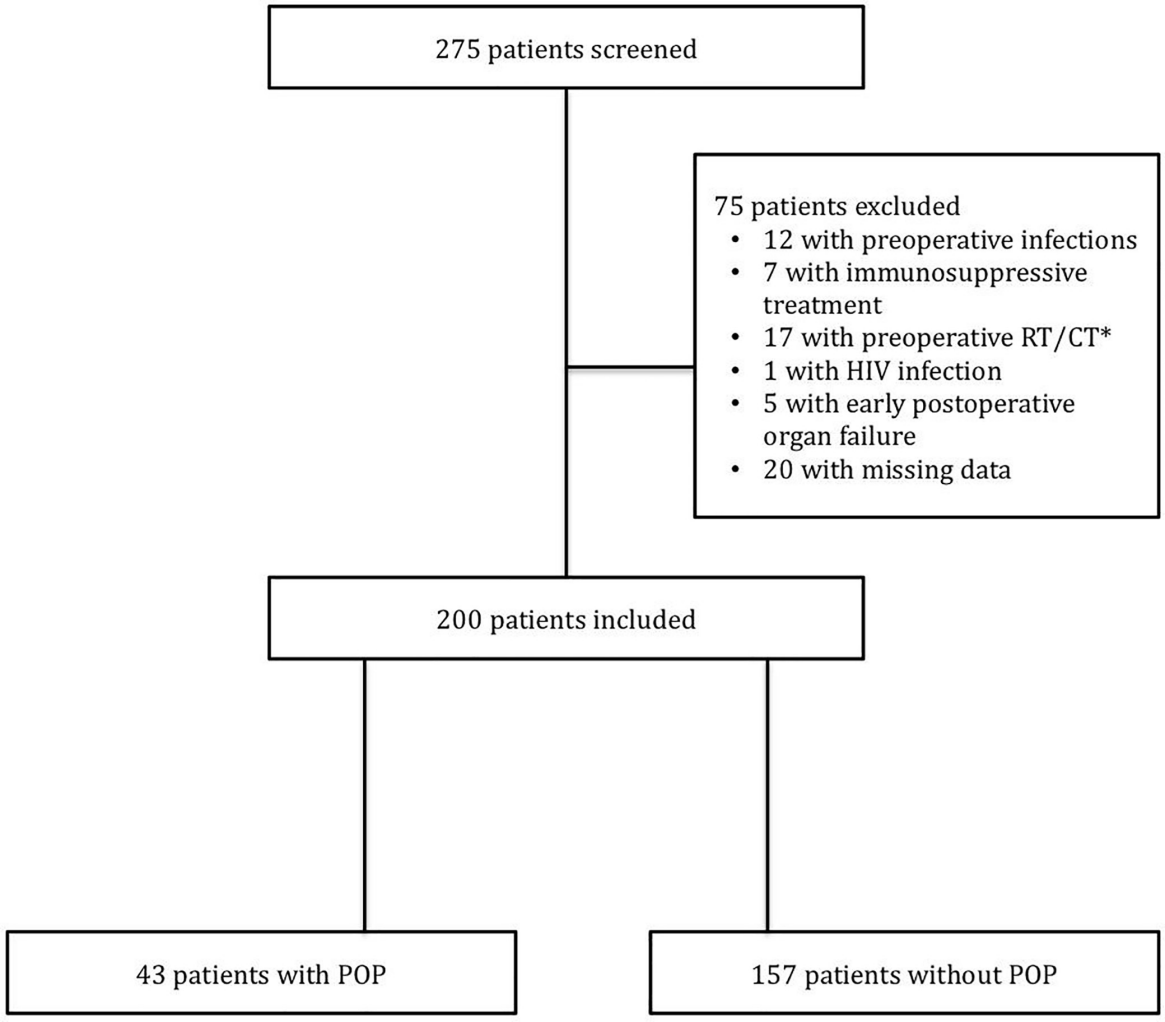
Flow chart of the study.

From January 2013 to May 2015, 275 patients were screened, of whom 75 were excluded and 200 were deemed to meet the inclusion criteria (Fig 1).

The demographic characteristics of these 200 patients are shown in Table 1. The mean operating time was 258 ± 59.4 minutes and the mean duration of mechanical ventilation was 283 ± 60 minutes. Thoracic epidural anesthesia was used for 29 patients (14.5%). The surgical procedure was thoracotomy, 156 (78%) patients undergoing lobectomy, 30 (15%) pneumonectomy and 14 (7%) bilobectomy. Antibiotic prophylaxis consistent with French recommendations was prescribed for all patients and comprised either cefazolin or clindamycin plus gentamicin the event of penicillin allergy. Fourteen patients had missing data: 3 patients with POP had not lymphocyte count the day before surgery, 10 patients with no postoperative infection had not lymphocyte count at postoperative day one and 1 patient had not information about BPCO, active smoking, NYHA and no lymphocyte count. The average of follow-up time for a patient was 5.9 ± 2.1 days.

**Table 1:** 

Characteristics	All patients	Patients with POP	Patients without POP n = 157	p
	n = 200	n = 43		
**Age, years, mean ± SD**	64 ± 11	66 ± 11	64 ± 11	0.09
**Sex**				0.117
Male, n (%)	149 (75)	36 (84)	113 (72)	
Female, n (%)	51 (25)	7 (16)	44 (28)	
**ASA score**				0.416
ASA 1–2, n (%)	90 (45)	17 (39)	73 (46)	
ASA 3–4, n (%)	110 (55)	26 (61)	84 (54)	
**BMI, mean ± SD**	25 ± 5			
**COPD, n (%)**	90 (45)	23 (55)	67 (43)	0.061
**Active smoker, n (%)**	63 (32)	25 (58)	38 (23)	0.062
**Chronic heart failure, n (%)**	64 (32)	21 (49)	43 (28)	0.024
(NYHA ≥ 2)				

POP = postoperative pneumonia; RT/CT = radiotherapy/chemotherapy

ASA: American Society of Anesthesiologists

COPD: Chronic Obstructive Pulmonary Disease

SD: Standard Deviation

### Patient characteristics

Forty-three patients (21.5%) developed POP at 2.5 ± 1.5 days post-surgery. The mean length of hospital stay was 12 ± 6 days and the 30-day mortality rate was 2.5% (7/275).

**Fig 2:**
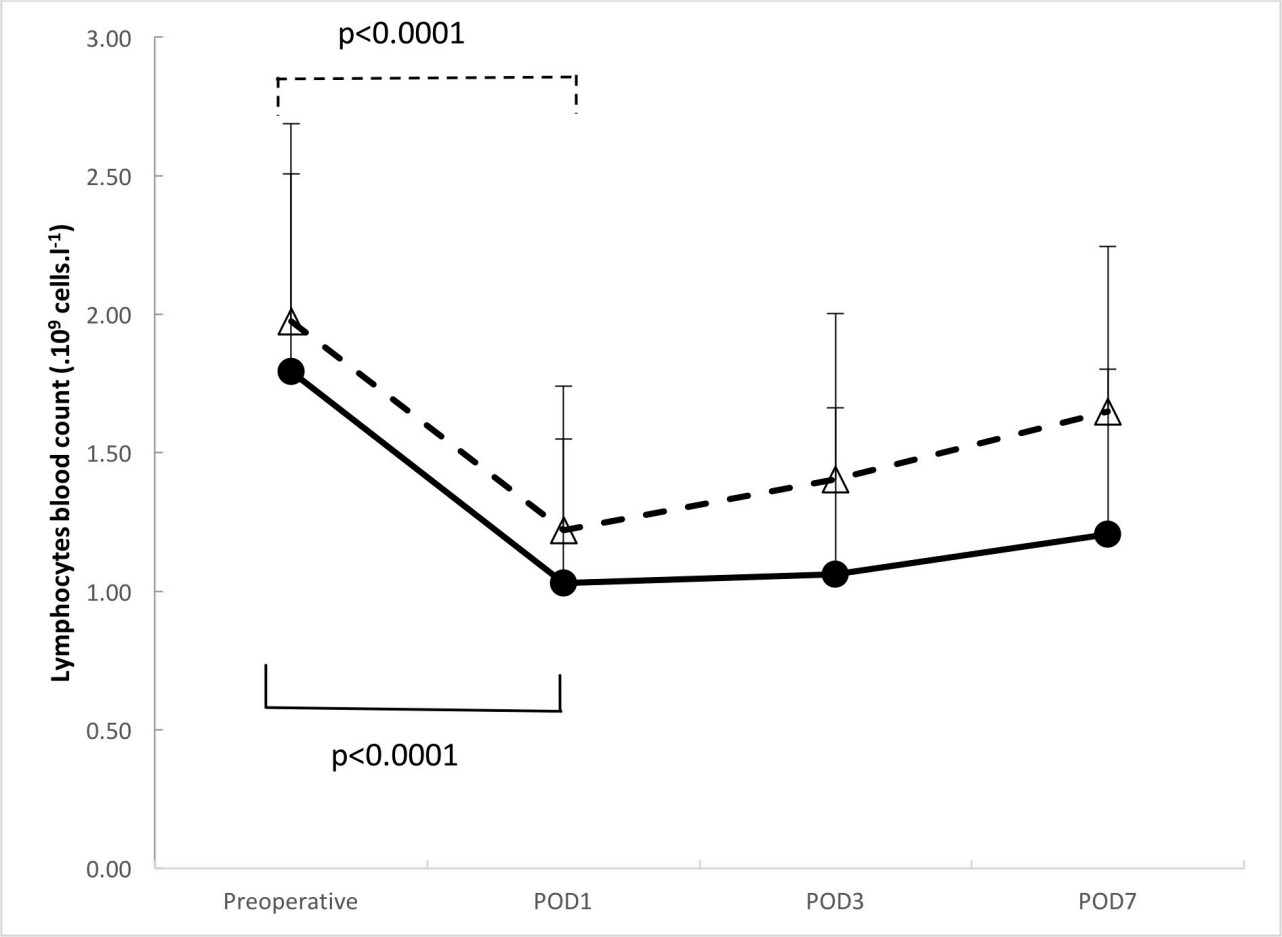
Changes of lymphocyte blood count. Black line with black circles shows patients’ lymphocyte counts with postoperative pneumonia. The dotted line with white triangles shows patients’ lymphocyte count with no postoperative pneumonia.

Mean preoperative lymphocyte counts were 2.0 ± 0.7 x 10^9^ cells/L and 1.8 ± 0.6 x 10^9^ cells/L in patients subsequently experiencing and not experiencing POP, respectively (*P* = .090), lymphocyte counts then declining to a nadir of 1.0 ± 0.5 x 10^9^ cells/L at postoperative day 1 (*P* < .0001 *versus* preoperative value) and 1.2 ± 0.5 x 10^9^ cells/L (*P* < .0001 *versus* preoperative value), respectively (Fig 2).

In multivariate analysis, postoperative lymphopenia at postoperative day 1 was the only variable related to lymphocyte count. It was significantly associated with an increased risk for POP after adjustments for ASA score ≥ 3, sex, age > 65 years old, COPD, active smoking and chronic heart failure (HR 2.09, 95% CI [1.01–4.29]) (Table 2).

**Table 2:** 

	Patients with POP	Patients without POP	Hazard Ratio	*P* value
	(n = 43)	(n = 157)	[95% CI]	
**Age > 65 years, n (%)**	26 (61)	65 (41)	Adjustment factor	
**Sex**				
**Male, n (%)**	36 (84)	113 (72)	Adjustment factor	
**Female, n (%)**	7 (16)	44 (28)		
			
**ASA ≥ 3, n (%)**	26 (61)	84 (54)	Adjustment factor	
**COPD, n (%)**	23 (55)	67 (43)	Adjustment factor	
**Active smoker, n (%)**	25 (58)	38 (23)	Adjustment factor	
**Chronic heart failure, n (%)**	21 (49)	43 (28)	Adjustment factor	
**Lymphocyte count at postoperative day 1**	1.0 ± 0.5	1.2 ± 0.5	2.09 [1.01–4.29]	0.046
**(x 10**^**9**^ **cells/L), mean ± SD**				
**Preoperative lymphocyte count**	1.8 ± 0.6	2.0 ± 0.7	1.39 [0.83–2.33]	0.211
**(x 10**^**9**^ **cells/L), mean ± SD**				
**Change in lymphocyte count from preoperation**	-42 ± 23	-36 ± 34	0.99 [0.97–1.01]	0.244
**to postoperative day 1 (%), mean ± SD**				

POD: Postoperative Day

POP: Postoperative Pneumonia

ASA: American Society of Anesthesiologists

COPD: Chronic Obstructive Pulmonary Disease

SD: Standard Deviation

### Multivariate analysis adjusted on currently described risk factors for postoperative pneumonia

Leukocyte and neutrophil responses to surgery are presented in the online supporting material (S1 and S2 Figs). Counts of these two cell lines did not differ between patients experiencing and not experiencing POP, either before surgery or at postoperative day 1.

**Fig 3:**
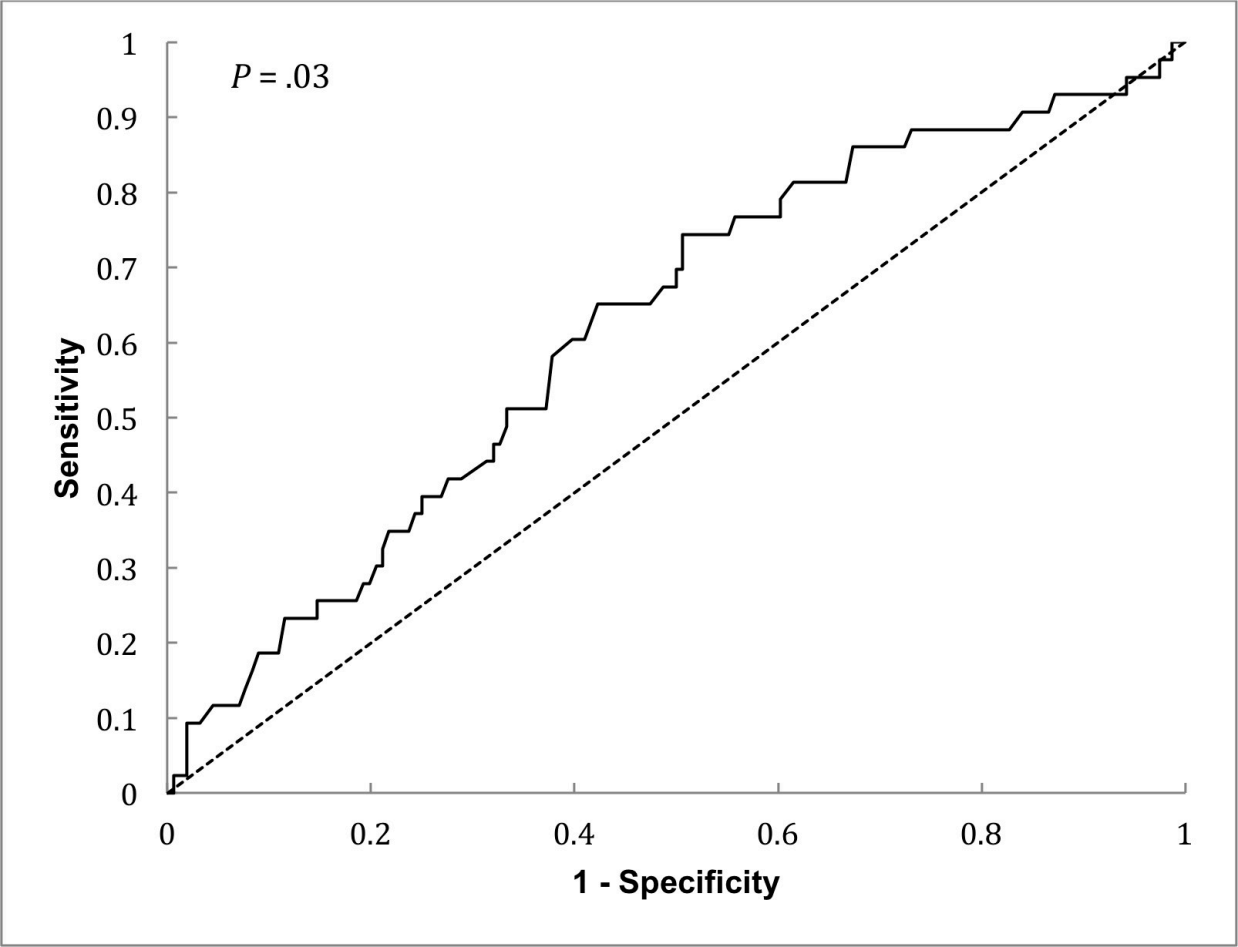
Receiver operating characteristic curve for the diagnosis of POP based on lymphopenia at postoperative day 1.

**Fig 4:**
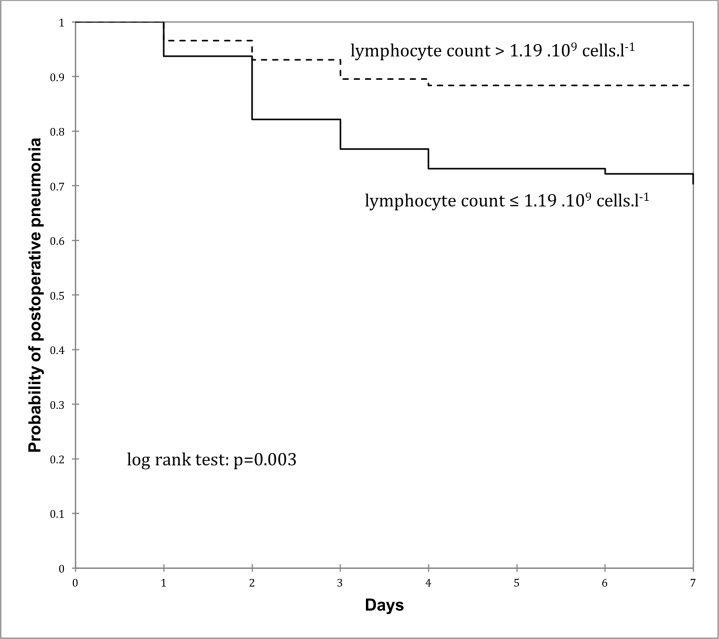
Kaplan-Meier curves showing the probability of postoperative pneumonia in patients with low or high lymphocyte count at postoperative day 1.

The ROC curve for the diagnosis of POP based on lymphopenia at postoperative day 1 is presented in Fig 3. Based on the value of Youden’s index (0.24), we have retained 1.19 x 10^9^ cells/L as the threshold for the diagnosis of POP (sensitivity = 74%, specificity = 49%, predictive positive value = 29%, predictive negative value = 88%). As shown in Fig 4, the POP rate at postoperative day 7 was higher in patients presenting a lymphocyte count ≤ 1.19 x 10^9^ cells/L at postoperative day 1 compared with patients in whom lymphocyte count was above this threshold (*P* = .003)

## Discussion

Our study highlighted in a multivariate analysis that postoperative lymphopenia at day 1 post-surgery is an independent risk factor for the development of pneumonia after elective surgery for lung cancer in multivariate analysis.

It is now recognized that stress-induced lymphopenia in patients exposed to trauma and sepsis is associated with the development of nosocomial infections. Gennari *et al*. showed that severe postoperative lymphopenia below a critical level (< 50% of the reference range) was an indicator of postoperative infection [13]. Similarly, in the context of spinal instrumentation surgery, Takahashi *et al*. reported data suggesting that lymphopenia (under 10% or 1.0 x 10^9^ cells/L) at 4 days post-surgery was predictive of surgical wound infection [14]. However, both these studies included limited numbers of patients (33 and 39 patients respectively). Moreover, the first study included both patients with malignant diseases and patients with non-malignant diseases [13], while the second study lacked details concerning the time to postoperative infection [14]. More recently, *Iwata et al*. did not demonstrate that the lymphocytes counts at days 4 and 7 postoperatively were a reliable biochemical marker predicting surgical site infection following instrumental spine fusion [15]. In addition, other risk factors for postoperative infections were not considered, generating a confounding effect. To the best of our knowledge, no study up to now has demonstrated in a multivariate analysis that surgery-induced lymphopenia is associated with postoperative infection. POP is a frequent complication, leading to increased morbidity and prolonged hospital stay. The development of POP after non-cardiac or lung cancer surgery is conditioned particularly by age, ASA class, COPD and smoking history in the year preceding the operation. In our study population, most of these risk factors were observed in patients presenting POP.

Interestingly, in our study population, the development of POP was associated with postoperative lymphopenia but not with absolute lymphocyte count before surgery or the absolute or percentage decrease in lymphocyte count after surgery. This result suggests that there may be a threshold value of lymphocyte count for risk of POP. Below this threshold, immune deficiency would favor secondary infections. However, the existence of such a threshold is still debated. Recently, Edwards *et al*. observed in a prospective study that preoperative lymphopenia, defined as a lymphocyte count ≤ 1.3 x 10^9^ cells/L or < 20% of the total leukocyte count, was associated with infectious complications in elective arthroplasty surgery [16]. The postoperative lymphopenia threshold determined in our retrospective single-center study cannot reasonably be retained as a cutoff value for the risk of POP. The existence and value of such a threshold should be confirmed in a large, prospective, multicenter study. The median time to onset of POP was 2.5 ± 1.5 days. As sepsis in itself can lead to lymphopenia [17] we did not take into account lymphocyte count beyond postoperative day 2 in the logistic regression model. Identify lymphopenia at postoperative day one must draw the attention of the clinician, in particular for patients with several risk factors of POP.

All of the patients included had thoracotomy and none had video-assisted thoracoscopic surgery (VATS). Increasingly VATS lobectomy are being performed and have been shown to be associated with reduced postoperative pulmonary complications in comparison to thoracotomy [18]. However, to date, there are conflicting data to conclude that the surgical approach is associated with a more or less severe lymphopenia [19–22]. This may limit the generalizability of our results.

The existence of postoperative lymphopenia has been recognized for many years [23], but has remained poorly described. The lymphocyte count nadir has been reported to occur as early as two hours after anesthesia induction or up to two days after surgery, recovery of lymphocyte count taking up to two weeks [23–29]. However, as already mentioned, the studies concerned were heterogeneous in terms of observed time to postoperative infection or the prevalence of cancer within the patient population included. In our study, the lymphocyte count nadir was observed at postoperative day 1 in patients both with and without POP. Similar times to lymphocyte count nadir have been previously described in the context of elective orthopedic surgery for arthroplasty, gastrointestinal surgery and neurosurgery [13,14,16]. Interestingly, the occurrence of pneumopathy did not seem to elicit a further decrease in lymphocyte count, but delayed recovery despite appropriate treatment.

In patients with cancer, particularly those with advanced disease, T lymphocyte homeostasis is impaired, leading to a loss of appropriate T lymphocyte responses [30]. Several mechanisms have been described to explain this T lymphocyte exhaustion and cancer-induced lymphopenia, including the presence of T lymphocytes expressing inhibitory products, increased numbers of regulatory T-cells and myeloid-derived suppressor cells. These changes have also been observed in patients presenting acute inflammation related to sepsis [30]. The mechanisms underlying postoperative lymphopenia have been less well characterized. However, some of the mechanisms described are similar to those identified in the context of sepsis and cancer. As surgery represents an aggression, we can hypothesize that some of the adverse changes in lymphocyte homeostasis observed following surgery are likely to be similar to those described in patients with sepsis and in cancer patients. Thus, a shift from Th1 helper cells to Th2 helper cells and increased numbers of regulatory T-cells have been shown to be involved in postoperative lymphopenia [24,31].

Our study has some limitations. We performed a multivariate analysis based on retrospective data. For this reason, our multivariate analysis was adjusted on the risk factors for POP most frequently described. POP is difficult to diagnose and the use of retrospective data may lead to overestimation of the rate of this complication. Our algorithm for the diagnosis of POP was similar to those previously used [2,4,6] and included chest X-rays interpreted by three experienced physicians in order to reduce these biases. In our study, 21.5% of patients developed POP, a rate consistent with those described in the literature [2,3]. The lymphopenia threshold defined in this study had a low sensitivity and specificity, probably due to a lack of statistical power.

## Conclusions

Our study showed that a low lymphocyte count at postoperative day 1 was independently associated with the development of pneumonia after lung cancer surgery. These results require to be confirm in a large prospective cohort.

## Supporting information

S1 Fig Changes in leukocyte count over time in patients who experienced (red line with red circles) and did not experience (blue line with blue triangles) postoperative pneumonia. POD = postoperative day.
(TIFF)

S2 Fig Changes in neutrophil count over time in patients who experienced (red line with red circles) and did not experience (blue line with blue triangles) postoperative pneumonia. (TIFF)
