# Supplementary figures and images for: Postoperative lymphopenia: An independent risk factor for postoperative pneumonia after lung cancer surgery, results of a case-control study
Source: PLoS One. 2018 Oct 15;13(10):e0205237. doi: 10.1371/journal.pone.0205237 (PMC6188898; doi:10.1371/journal.pone.0205237)

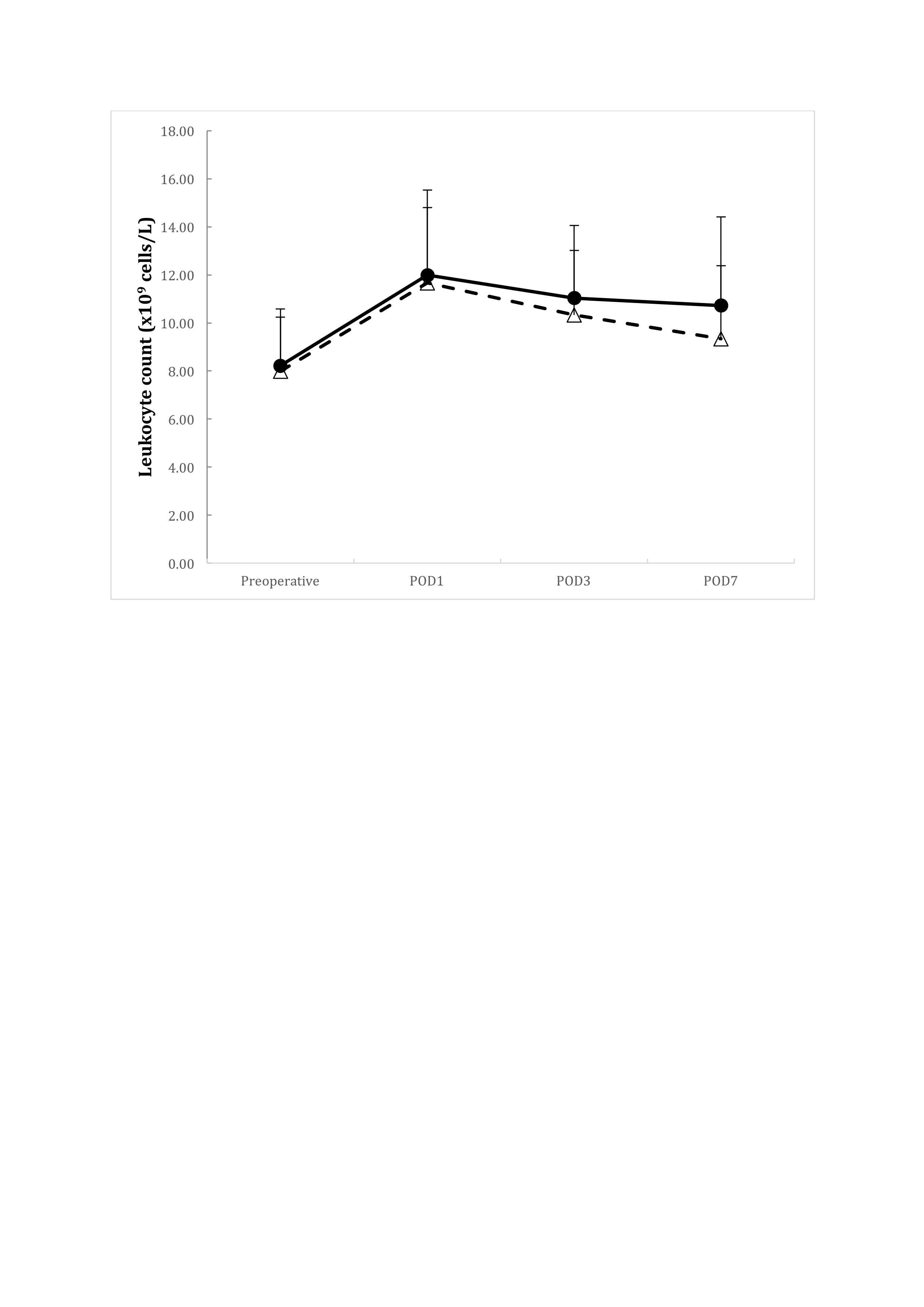

Supplement: S1 Fig — POD = postoperative day. (TIFF) [file pone.0205237.s001.tiff]

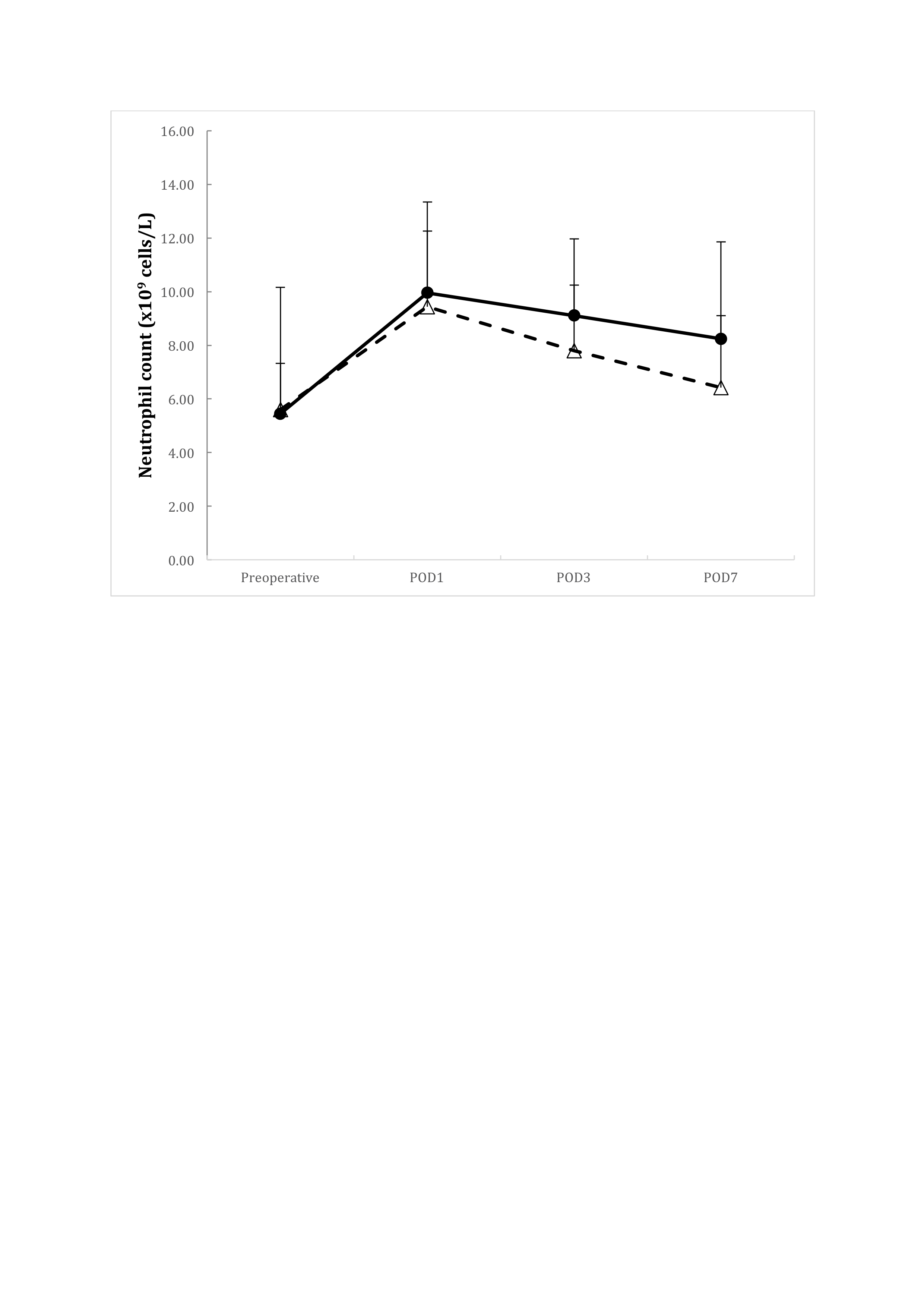

Supplement: S2 Fig — (TIFF) [file pone.0205237.s002.tiff]
